# PIRCHE-II: an algorithm to predict indirectly recognizable HLA epitopes in solid organ transplantation

**DOI:** 10.1007/s00251-019-01140-x

**Published:** 2019-11-18

**Authors:** Kirsten Geneugelijk, Eric Spierings

**Affiliations:** Laboratory of Translational Immunology, University Medical Center Utrecht, Heidelberglaan 100, 3584 CX Utrecht, The Netherlands

**Keywords:** HLA, Histocompatibility, Transplantation, Matching, PIRCHE-II, Epitopes, Solid organ transplantation

## Abstract

Human leukocyte antigen (HLA) mismatches between donors and recipients may lead to alloreactivity after solid organ transplantation. Over the last few decades, our knowledge of the complexity of the HLA system has dramatically increased, as numerous new HLA alleles have been identified. As a result, the likelihood of alloreactive responses towards HLA mismatches after solid organ transplantation cannot easily be assessed. Algorithms are promising solutions to estimate the risk for alloreactivity after solid organ transplantation. In this review, we show that the recently developed PIRCHE-II (Predicted Indirectly ReCognizable HLA Epitopes) algorithm can be used to minimize alloreactivity towards HLA mismatches. Together with the use of other algorithms and simulation approaches, the PIRCHE-II algorithm aims for a better estimated alloreactive risk for individual patients and eventually an improved graft survival after solid organ transplantation.

## Introduction

The development of human leukocyte antigen (HLA) antibodies directed towards HLA mismatches between donor and recipient is a major cause of allograft rejection after solid organ transplantation (Everly et al. 2013; Terasaki and Cai 2008; Zhang et al. 2005). One of the approaches to avoid these alloreactive humoral responses is to limit the number of HLA mismatches between donor and recipient (Ansari et al. 2014; Susal and Opelz 2013). Previous research has shown that better HLA matching indeed resulted in a reduced need for immunosuppressive treatment as well as less allograft rejection after kidney transplantation (Susal and Opelz 2013). Therefore, organ exchange organizations, such as Eurotransplant, have implemented the HLA matching factor in their allocation strategy (Doxiadis et al. 2004; Persijn 2006), thereby aiming for transplanting kidneys with low numbers of HLA mismatches between donors and recipients (Susal and Opelz 2013). Although limiting the number of HLA mismatches between donor and recipient is an effective method to reduce the risk for kidney allograft rejection, this approach has some limitations. First, for some patients, it may be more difficult to select donors with a low number of HLA mismatches due to the ethnic background of the patient. Second, previous studies have shown that HLA mismatches do not equally contribute to alloreactivity (Claas et al. 2005; Doxiadis et al. 1996). Some HLA mismatches may have an immunological impact and will elicit severe alloreactivity, whereas others may have not an immunological impact and, therefore, will not elicit alloreactivity (Claas et al. 2005; Doxiadis et al. 1996). Therefore, instead of counting the number of HLA mismatches between donor and recipient, one should preferable transplant with those HLA mismatches that will not lead to alloreactivity, whereas transplantation with HLA mismatches that will lead to alloreactivity should be avoided.

With the current HLA allele database reporting more than 18,000 protein variants (IPD-IMGT/HLA 3.37, July 10, 2019, https://www.ebi.ac.uk/ipd/imgt/hla/stats.html, visited August 13, 2019), in vitro or in vivo assessment of the antigenicity and immunogenicity of each individual HLA mismatch combination is challenging. Rather than testing the antigenicity and/or immunogenicity of HLA mismatches individually, in silico algorithms may provide an alternative solution. Several algorithms have been developed over the past decades, aiming to predict alloimmune reactivity in general. Over the past few years, the application of these algorithms in the context of solid organ transplantation has been investigated (Geneugelijk et al. 2014). Indeed, there is ample evidence that in silico algorithms are able to predict the risk for HLA antibody formation after solid organ transplantation. In this review, we will briefly describe the rational behind these developed algorithms and we will discuss in more detail the recently developed PIRCHE-II (Predicted Indirectly ReCognizable HLA Epitopes) algorithm, which can estimate the risk for developing alloreactivity towards HLA mismatches. Eventually, the use of these algorithms in solid organ transplantation may lead to a better estimation of the immunological risk for allograft rejection.

## Preventing alloreactivity towards HLA by epitope-based HLA matching

A better understanding of alloreactive responses in solid organ transplantation leading to graft rejection formed the basis of predicting the immunogenicity of HLA mismatches. Over the past decades, not only the number of identified HLA alleles has been increased, but also their exact amino acid sequence and their three-dimensional structure have been extensively unraveled. As the amino acid sequence and three-dimensional structure of HLA molecules became available, epitopes on HLA that are involved in the humoral alloreactive response after solid organ transplantation were identified (Dankers et al. 2004b; Kosmoliaptsis et al. 2008). Epitopes are parts of foreign HLA molecules that can be recognized by the immune system. Some of these epitopes may be present on multiple different HLA antigens (Dankers et al. 2004b). Epitopes that are present on HLA of both donor and recipient will not elicit alloreactive responses, whereas epitopes that are mismatched between donor and recipient may elicit alloreactive responses. Therefore, one of the approaches to estimate the clinical impact of individual HLA mismatches on alloreactivity may be to quantify the total epitope load between donor and recipient, thus by counting the number of mismatched epitopes between donor and recipient (Duquesnoy 2008; Kosmoliaptsis et al. 2008). As the total epitope load may provide more information on the potential clinical consequences of HLA mismatches, this approach enables a more precise determination of the donor-recipient compatibility compared to counting the total number of HLA mismatches (Duquesnoy et al. 2008b; Kosmoliaptsis et al. 2008). The identification of these epitopes has led to a concept called epitope-based HLA matching: a matching strategy in which epitopes are used for HLA matching rather than counting the number of HLA mismatches (Claas and Heidt 2017; Duquesnoy 2016; Duquesnoy 2017).

Initially, epitopes that are involved in the humoral immune response after solid organ transplantation were considered as an approach for epitope-based HLA matching. First, epitopes involved in the humoral immune response were predicted via the HLAMatchmaker algorithm (Duquesnoy 2002; Duquesnoy 2006; Duquesnoy 2008). The epitopes identified via the HLAMatchmaker algorithm are designated as eplets. Eplets are small amino acid polymorphisms on the external domains of HLA molecules, which are different between donor and recipient (Duquesnoy 2002; Duquesnoy 2006; Duquesnoy 2008). Only amino acid polymorphisms that are accessible to HLA antibodies due to their location on the HLA molecule are considered eplets (Duquesnoy 2002; Duquesnoy 2006; Duquesnoy 2008). Thus, the HLAMatchmaker algorithm considers each HLA molecule as a set of epitopes/eplets and only eplets that differ between donor and recipient are considered as an alloreactive target. Considerable research has been devoted to investigating the impact of the number of eplet mismatches between donor and recipient on HLA antibody formation and graft survival. Patients who were transplanted with a higher number of mismatched eplets have a higher risk for donor-specific HLA antibody formation after transplantation (Daniels et al. 2018; Duquesnoy et al. 2008a; Kubal et al. 2018; McCaughan et al. 2018; Walton et al. 2018; Wiebe et al. 2013). Moreover, additional studies suggest that matching based on the number of eplets may lead to an improved graft outcome in kidney (Wiebe et al. 2013), heart (Sullivan et al. 2015), lung (Walton et al. 2016), liver (Ekong et al. 2019), and cornea transplantation (Bohringer et al. 2010).

## The role of T cell-mediated alloreactivity in humoral HLA responses

Although the relation between HLAMatchmaker eplets and humoral alloreactive responses was studied extensively over the past years, the alloreactive immune responses after solid organ transplantation are not only limited to the B cells, but also comprise T cell-mediated alloreactivity. T cells can interact with allogeneic HLA via three different pathways: the direct T cell recognition pathway, the indirect T cell recognition pathway, and the semi-direct T cell recognition pathway (Ali et al. 2013; Game and Lechler 2002; Marino et al. 2016). The antigenic ligand for recognition is different for each of these three pathways. Recognition of complete allogeneic HLA molecules located on the surface of allogeneic cells is essential for direct T cell recognition, whereas T cell recognition of mismatched HLA-derived epitopes presented by HLA of non-allogeneic cells characterizes indirect T cell recognition (Ali et al. 2013; Game and Lechler 2002; Marino et al. 2016). Thus, indirect T cell recognition depends on processing and presentation of mismatched HLA by non-allogeneic cells, whereas this is not the case for direct T cell recognition. Alternatively, allogeneic HLA may be recognized by T cells in a semi-direct fashion; these T cells recognize complete allogeneic HLA:peptide complexes that are transferred from allogeneic cells to non-allogeneic antigen-presenting cells (Ali et al. 2013; Marino et al. 2016). Although of a different origin, the cognate interaction between the alloimmune T cell receptor and its ligand is again either via the intact allogeneic HLA protein on the cell surface or via a non-self HLA-derived peptide presented in self HLA (Marino et al. 2016). The underlying processes and characteristics thus follow those of the direct and indirect recognition pathways, respectively.

Indirect T cell recognition of HLA mismatches may result in allograft rejection after solid organ transplantation in two ways. Type 1 CD4+ T-helper cells may, after indirect recognition of mismatched HLA presented by a recipient antigen-presenting cell, provide help to CD8+ T cell responses, which may cause graft damage (Ali et al. 2013). However, both direct pathway CD4+ T cells and indirect pathway CD4+ T cells are able to provide help to CD8+ cytotoxic T cells (Marino et al. 2016), and literature suggests that the role of the indirect pathway in providing help to CD8+ T cells may be limited (Ali et al. 2013; Marino et al. 2016). Indirect CD4+ T cells seem to play a more pronounced role in humoral alloreactivity (Ali et al. 2013; Marino et al. 2016). Via the indirect recognition pathway, follicular CD4+ T-helper T cells can provide help to B cells, which is required for a humoral alloreactive response (Badell and Ford 2016; Conlon et al. 2012; de Graav et al. 2015; Steele et al. 1996). In this process of providing help, mismatched HLA is internalized and processed in B cells of the recipient. The HLA-mismatched derived peptides arised from this processing can subsequently be presented to T cells via HLA class II molecules on the cell surface. Upon indirect recognition of these HLA-mismatched derived epitopes, T cells can provide help to B cells, leading to B cell proliferation, differentiation of naive B cell into memory B cells and plasma cells, and IgM to IgG isotype switching (Steele et al. 1996). Thus, to form IgG HLA-specific antibodies, B cells need to be activated by CD4+ T cells. In this process of HLA antibody formation, the B cell and the CD4+ T cell respond to potential different epitopes that are located on the same antigen, a phenomenon called linked recognition (Mitchison 2004).

The pivotal role of indirect T cell recognition in HLA antibody formation was shown in various studies (Conlon et al. 2012; Lovegrove et al. 2001; Sauve et al. 2004; Steele et al. 1996). In mice, IgG isotype HLA antibodies were only produced via the indirect T cell recognition of allogeneic HLA (Sauve et al. 2004). These data suggest that indirect CD4+ T cell recognition is an essential factor in the production of isotype-switched donor-specific HLA antibodies. Consequently, indirect CD4+ T cell recognition of mismatches HLA may thereby impact graft function after solid organ transplantation. Indeed, the hypothesis that indirect T cell recognition plays a role in the development of allograft rejection after solid organ transplantation is supported by various studies. For example, indirect T cell allorecognition has been shown to impact both acute and chronic allograft rejection in clinical organ transplantation and experimental organ transplantation models (Benichou et al. 1992; Fangmann et al. 1992; Lee et al. 1994). Furthermore, indirect T cell allorecognition has a role in kidney (Baker et al. 2001; Vella et al. 1997), heart (Hornick et al. 2000; Lee et al. 2001; Suciu-Foca et al. 1998), and lung (SivaSai et al. 1999) allograft rejection after transplantation.

## Epitope-based matching based on predicting indirect T cell recognition

Since indirect T cell alloreactivity after solid organ transplantations plays such an important role in the establishment of a humoral response towards HLA, identification of the mismatched HLA-derived epitopes that can be recognized by T cells via the indirect pathway may be an alternative approach for epitope-based matching. On that basis, the PIRCHE algorithm was established to identify T cell epitopes. In contrast to the HLAMatchmaker eplets, these T cell epitopes are the epitopes present on mismatched HLA that are indirectly recognized by T cells (Geneugelijk et al. 2014). In the context of solid organ transplantation, the algorithm uses in silico antigen presentation pathway estimations to predict the number of HLA mismatch-derived peptides that can be presented in the context of recipient HLA class II (Geneugelijk et al. 2015; Geneugelijk et al. 2014). As these peptides are presented on recipient HLA class II molecules, these peptides are designated PIRCHE-II. Their appearance in the groove of HLA class II is assumed to promote helper T cell responses, either leading to a better T cell response (via type 1 CD4+ T-helper cells) or a mature humoral response (via follicular CD4+ T-helper T cells). Due to the described role of indirect T cell recognition pathway in humoral alloreactivity, PIRCHE-II are likely predominantly involved in the production of HLA antibody formation, thereby indirectly affecting graft function of the transplanted organ. Thus, for each individual donor-recipient couple, the T cell epitope-load or number of PIRCHE-II can be calculated, which theoretically reflects the level of CD4+ T cell alloreactivity. A higher number of PIRCHE-II is likely associated with a high level of CD4+ T cell alloreactivity, whereas a low number of PIRCHE-II is likely associated with a low level of CD4+ T cell alloreactivity.

## What are the technical challenges of the PIRCHE-II algorithm?

The PIRCHE-II algorithm can predict whether mismatched HLA-derived peptides are able to bind to HLA class II molecules, thereby serving as a target for indirect T cell recognition (Geneugelijk et al. 2014). For these predictions, the PIRCHE-II algorithm uses an algorithm designated as NetMHCIIpan (Geneugelijk et al. 2014; Karosiene et al. 2013; Nielsen et al. 2010). Only peptides with a sufficient predicted binding affinity for HLA class II molecules are designated a PIRCHE-II. The predicted binding affinity is defined sufficient when peptides have an binding affinity of IC50 < 1000 nM for HLA class II molecules (Geneugelijk et al. 2014; Southwood et al. 1998). After counting the number of mismatched HLA-derived predicted HLA class II binders, these HLA binders are divided into self and non-self peptides (Geneugelijk et al. 2014). Self peptides are the mismatched HLA-derived predicted HLA class II binders that are present in both HLA of the donor and in HLA of the recipient, whereas non-self peptides are uniquely present in HLA of the donor. Since only non-self peptides are epitopes able to induce a clinically relevant T cell response, only these non-self peptides are counted as PIRCHE-II (Geneugelijk et al. 2014). These non-self peptides should at least differ in one amino acid with the HLA of the recipient in order to be counted as a PIRCHE-II (Geneugelijk et al. 2014). Thus, PIRCHE-II are those peptides that are relevant HLA class II binders and that contain at least one single amino acid difference between donor and recipient.

The fact that, theoretically, already a single amino acid difference between donor and recipient may lead to a PIRCHE-II indicates that complete amino acid sequences of HLA molecules of donor and recipient are required to be able to calculate the number of PIRCHE-II for specific donor-recipient couples. This aspect is quite challenging, as the complete amino acid sequence is lacking for the majority of the identified HLA alleles. Both DNA and amino acid sequences of all worldwide identified HLA alleles are stored in the IMGT/HLA database (available via: http://www.ebi.ac.uk/ipd/imgt/hla/ (Robinson et al. 2015; Robinson et al. 2016)). However, the HLA amino acid sequences in the IMGT/HLA database are predominantly limited to the extracellular domains of the HLA proteins, whereas the HLA amino acid sequences of intracellular domains are often lacking (Geneugelijk et al. 2016). Our previous study investigating the IMGT/HLA database of 2015 showed that only a tenth of all identified HLA alleles at that time (*n* = 9577 HLA class I alleles and 2591 HLA class II alleles at two field-resolution HLA sequences) were completely present in the IMGT/HLA database (Geneugelijk et al. 2016). Since PIRCHE-II can be derived from the complete HLA protein and not only from the extracellular domains of the HLA protein, we developed an automated homology-based nearest neighbor approach (Geneugelijk et al. 2016) to extend the incomplete amino acid HLA sequences present in the IMGT/HLA database. Although this approach may introduce a limited amount of errors, most of the sequences can be reliably predicted (Geneugelijk et al. 2016). Nevertheless, submitting complete amino acid sequences to the IMGT/HLA database is still required to avoid amino acid mispredictions and, consequently, PIRCHE-II mispredictions. To further improve the quality of our amino acids sequences extensions, we regularly repeat this homology-based nearest neighbor approach also by implementing newly submitted complete amino acid sequences. Further validation studies to investigate whether implementation of these newly submitted complete amino acid sequences into the approach do lead to a more reliable amino acid prediction are currently ongoing

Although we have developed a method to extend incomplete HLA amino acid sequences using the automated homology-based nearest neighbor approach, one of the major challenges in determining the amino acid differences between donor and recipient is the lack of HLA typing information of donors and recipients. Preferably, two-field resolution HLA typing is required of both donor and recipient to determine the amino acid differences between donor and recipient. High-resolution HLA typing of deceased solid organ transplantation donors is especially challenging, due to the limited time that is available to perform HLA typing. Consequently, high-resolution HLA typing is often unavailable for deceased donors. Several methods have been sought to allow high-resolution HLA typing within a reasonable timeframe, such as minION (Goodwin et al. 2015), a third generation sequencing technology of Oxford Nanopore technologies, which shows an increasing sequencing accuracy (Carapito et al. 2016; Duke et al. 2019; Liu et al. 2018). Alternatively, for cases where the minION technology cannot be used in daily practice, we developed an additional computational method in 2017 to be able to calculate the number of PIRCHE-II using serological split level HLA typing (Geneugelijk et al. 2017). This method uses serological split HLA typing and HLA haplotype frequency tables of the National Marrow Donor Program to determine all potential high-resolution HLA typings that may correspond to a given serological split HLA typing. Thus, for every serological split level typing of donor and recipient, a list of all potential high-resolution HLA typings is generated. After identifying all potential high-resolution HLA typings from the serological split level HLA typing, PIRCHE-II is calculated for each of the potential high-resolution HLA typings of both donor and recipient. Since the likelihood of high-resolution HLA typing may vary between different potential high-resolution HLA typings, the PIRCHE-II values are subsequently weighted by the haplotype frequency of the high-resolution HLA typing in the general population. Via this approach, PIRCHE-II values calculated based on a high-resolution HLA genotype that is frequently present in the general population will contribute more to the final PIRCHE-II number compared to PIRCHE-II values calculated from a high-resolution HLA genotype that is less frequently present in the general population. A validation study showed that this approach can be used to reliably predict the number of PIRCHE-II for the majority of the donor-recipient couples when high-resolution HLA typing is unavailable (Geneugelijk et al. 2017). The predictions further improved when high-resolution HLA typing of the patient and serological split level HLA typing of the donor was used (Geneugelijk et al. 2017). Thus, although two-field resolution HLA typing is still preferred to calculate the number of PIRCHE-II, the number of PIRCHE-II can be predicted in a reliable manner for a majority of the donor-recipient couples when two-field resolution HLA typing data is unavailable (Geneugelijk et al. 2017). However, sharing next-generation sequencing-based genotype datasets internationally combined with ethnical data allows the establishment of more reliable HLA haplotype frequency tables and, consequently, will further lead to a more reliable prediction of the PIRCHE-II number.

In addition to the technical challenges of the PIRCHE-II algorithm, some challenges still remain. First, the PIRCHE-II studies performed thus far have been focusing on PIRCHE-II presented by HLA-DRB1, whereas PIRCHE-II can also be presented on HLA-DQA1;HLA-DQB1 heterodimers, HLA-DPA1;HLA-DPB1 heterodimers, and HLA-DRB3/4/5. Recently, PIRCHE version 3.0 became available, which also has implemented PIRCHE-II presentation by HLA-DQ, HLA-DP, and HLA-DRB3/4/5. Further studies will indicate whether PIRCHE-II presented by these additional HLA class II loci will correlate with clinical outcome. Second, PIRCHE-II are theoretical predicted epitopes. Since the antigen processing pathways for HLA class II presentation remains elusive, no algorithms currently exist to predict antigen processing for the HLA class II presentation pathway (Mettu et al. 2016). Therefore, it may well be the case that some of the PIRCHE-II are predicted to bind to HLA class II molecules, whereas these peptides cannot be generated biologically by the antigen processing pathway. This aspect suggests that we may overestimate the genuine number of PIRCHE-II for certain donor-recipient couples. Furthermore, in our current PIRCHE-II model, all individual identified PIRCHE-II are currently counted as equal with regard to their immunological weight. Thus, one PIRCHE-II is considered to contribute equally to the immune response compared to another PIRCHE-II. This, however, might not be the case, as several aspects may be contributing to the immunological impact of an individual PIRCHE-II. For example, since individual HLA loci and even different HLA alleles within a certain HLA locus may be expressed differently (Fernandez-Vina et al. 2013; McCutcheon et al. 1995; Petersdorf et al. 2014), it is expected that lower expressed HLA alleles are less likely to be presented as a PIRCHE-II. In accordance, it is also expected that PIRCHE-II presented by lower expressed HLA class II alleles are less likely to be recognized by T cells. In addition to the differential HLA expression, the immunological impact of PIRCHE-II is also determined by the PIRCHE-II:T cell receptor interaction. Some of the amino acid positions of the presented peptide seem to contribute more to T cell receptor interaction than others (De Oliveira et al. 2000; Rudolph et al. 2006), indicating that the amino acid positions of the PIRCHE-II that interact with the T cell receptor may have a higher impact of the immunogenicity than the amino acid positions that do not directly interact with the T cell receptor. Thus, further studies are required to investigate whether individual PIRCHE-II indeed may impact the immunological response differentially and, consequently, the PIRCHE-II number should be weighted according to their individual immunological impact.

## The impact of PIRCHE-II on HLA antibody formation in solid organ transplantation

Since indirect CD4+ T cells play a role in the development of HLA antibodies (Steele et al. 1996), the role of PIRCHE-II in HLA antibody formation in solid organ transplantation and other HLA sensitization settings was investigated. First, PIRCHE-II presented by HLA-DRB1 was shown to be involved in de novo donor-specific HLA antibody formation after transplantectomy in kidney transplantation recipients (Otten et al. 2013). However, when the HLA-DRB1 background of the recipient was scrambled and PIRCHE-II numbers were recalculated using the different HLA-DRB1 background, the relation between PIRCHE-II numbers and de novo donor-specific HLA antibody formation was absent (Otten et al. 2013). This observation indicates that the HLA-DRB1 background of the recipient is essential for the immunogenicity of HLA mismatches. Previous studies are in line with this observation by showing that the HLA-DR background of the recipients is highly correlated with the production of HLA antibodies towards specific HLA class I antigens and against the Bw4 epitope (Dankers et al. 2004a; Fuller and Fuller 1999). Since the role of PIRCHE-II in de novo donor-specific HLA antibody formation after transplantectomy was studied in a relatively small cohort (*n* = 21 kidney transplantation recipients), additional studies were performed to confirm this observation. In a cohort of 301 mother-child pairs, PIRCHE-II presented by HLA-DRB1 was related to maternal HLA antibody formation directed towards mismatched HLA of the child (Geneugelijk et al. 2015). HLA mismatches between mother and child that yield a higher number of PIRCHE-II had an increased probability of HLA antibody formation compared to HLA mismatches that yield a low number of PIRCHE-II (Geneugelijk et al. 2015). Thus, this study confirms that PIRCHE-II is related to HLA antibody formation in general.

In a large German cohort consisting of 2787 kidney transplantation recipients, the role of PIRCHE-II on donor-specific HLA antibody formation was further studied (Lachmann et al. 2017). Besides the observation that both PIRCHE-II numbers and HLAMatchmaker eplet numbers were related to de novo donor-specific HLA antibody formation, multivariate analyses also showed that PIRCHE-II was independent predictor for donor-specific HLA antibody formation (Lachmann et al. 2017). This large study also allowed investigating the role of PIRCHE-II in locus-specific de novo HLA antibody formation, as the previously described studies only investigated the role of HLA class I-derived PIRCHE-II in donor-specific HLA antibody formation (Geneugelijk et al. 2015; Otten et al. 2013). With higher PIRCHE-II numbers, the probability for de novo donor-specific HLA antibody formation was increased against predominantly HLA-DRB1, and -DQ mismatches, and to a lesser extent against HLA-A and -B mismatches (Lachmann et al. 2017). In an additional cohort consisting of 36 kidney transplantation recipients, the impact of PIRCHE-II on locus-specific de novo HLA antibody formation was further confirmed (Daniels et al. 2018). This rather small multicenter study focused on impact of HLA class II-derived PIRCHE-II on de novo donor-specific HLA antibody formation. In accordance with the German study, this study showed that the occurrence of de novo donor-specific HLA-DQB1 antibodies after kidney transplantation is related to the number of HLA-DQB1-derived PIRCHE-II (Daniels et al. 2018). Thus, these studies suggest that PIRCHE-II impacts donor-specific HLA antibody formation after kidney transplantation.

Although considerable research has been devoted to studying the role of PIRCHE-II in donor-specific HLA antibody formation after kidney transplantation, less research has been devoted to studying the role of PIRCHE-II in donor-specific HLA antibody formation in other solid organ transplantation settings. For pancreas and pancreatic islet transplantation, the role of PIRCHE-II in HLA antibody formation was studied in a small cohort, consisting of 26 pancreas transplantation recipients and 18 pancreatic islet transplantation recipients (Chaigne et al. 2016). Although HLA class I-derived PIRCHE-II was not associated with the development of HLA class I-specific antibodies, this study indicates that HLA class II-derived PIRCHE-II impact the development of HLA class II-specific antibodies in these patients (Chaigne et al. 2016). In a more recent study, PIRCHE-II was also studied in the context of pediatric intestinal and multivisceral transplantation (Talayero et al. 2018). In these transplantation settings, the impact of donor-specific HLA antibodies on transplant outcome is still under debate (Talayero et al. 2018). This study showed that the number of epitope mismatches, both HLAMatchmaker eplets and PIRCHE-II, were not associated with de novo donor-specific HLA antibody formation (Talayero et al. 2018). With regard to liver transplantation, in a cohort of 379 patients who underwent their first liver transplantation between 2000 and 2017, PIRCHE-II numbers were higher among patients who developed de novo donor-specific HLA antibodies compared to patients who did not develop de novo donor-specific HLA antibodies (Meszaros et al. 2019). Moreover, an additional analysis showed that the impact of PIRCHE-II on de novo donor-specific HLA antibody formation was most prominent for HLA-DRB1 and -DQB1 mismatches (Meszaros et al. 2019). Thus, these studies suggest that PIRCHE-II also may play a role in different transplantation settings than kidney transplantation. However, since the number of studies are limited, performed in a limited number of transplantation settings, and often performed in small heterogeneous study cohorts, further validations of these observations are of utmost importance before drawing firm conclusions.

## The impact of PIRCHE-II on allograft survival in solid organ transplantation

The majority of the studies thus far have been focusing on the role of PIRCHE-II on donor-specific HLA antibody formation in different transplantation settings. Due to the close relation between de novo donor-specific HLA antibody formation and allograft rejection, the relation between PIRCHE-II and allograft rejection was further investigated in two large cohorts of kidney transplantation recipients. In a cohort of 2918 kidney transplantation recipients who were transplanted in the Netherlands between 1995 and 2005, we showed that, adjusted for confounders, high PIRCHE-II numbers were related to allograft rejection (Geneugelijk et al. 2018). This association was most prominent in patients receiving their first kidney transplantation. In a parallel study, Lachmann et al. reported an impact of PIRCHE-II numbers on allograft survival (*n* = 2787 kidney transplantation recipients) (Lachmann et al. 2017). Thus, these studies indicate that PIRCHE-II is related to allograft rejection in kidney transplantation. Whether PIRCHE-II also relates to allograft rejection in other forms of transplantation warrants further investigation.

## How to use the PIRCHE-II algorithm in the clinic?

Cumulating evidence suggests that algorithms are a better manner to predict alloreactivity after solid organ transplantation than counting the number of HLA mismatches. Multiple studies showed that HLAMatchmaker eplets are related to HLA antibody formation (Dankers et al. 2004b; Wiebe et al. 2013), and in this review, we showed that also the PIRCHE-II numbers are related to de novo donor-specific HLA antibody formation after transplantation. Since both HLAMatchmaker eplets and T cell epitopes are required to establish a proper alloreactive response, both algorithms are preferable clinically used in parallel, as both are predicting differential immunogenic epitopes. Indeed, in our study among pregnant women, we already showed that the number of PIRCHE-II did not correlate with the number of HLAMatchmaker eplets (Geneugelijk et al. 2015). Lachmann et al. did find a moderate correlation between the number of PIRCHE-II and the number of HLAMatchmaker eplets, although this correlation was not found for every HLA mismatch (Lachmann et al. 2017). Moreover, the latter study showed that the PIRCHE-II algorithm and the HLAMatchmaker algorithm are two independent predictors of de novo donor-specific HLA antibody formation (Lachmann et al. 2017). These results suggest that both algorithms predict different epitopes that are located on the same mismatched HLA, which is in line with the concept of linked recognition, and that both algorithms should be taken into account to estimate the alloreactive potential of a certain HLA mismatch.

As the current golden standard in predicting alloreactivity is still counting the number of mismatches between donor and recipient, implementation of algorithms in the donor-selection procedure for solid organ transplantation requires a shift in these old standards of HLA matching in transplantation. PIRCHE-II can be implemented into the donor-selection procedure in two ways. First, PIRCHE-II numbers for a certain donor-recipient combination may indicate the risk level for allograft rejection. Patients who are at risk for allograft rejection based on high PIRCHE-II numbers can be more closely monitored after transplantation in order to be able to treat potential allograft rejection on time. However, the development of de novo donor-specific HLA antibodies cannot be prevented via this method. To minimize the risk for developing de novo donor-specific HLA antibodies after transplantation, patients should preferable be transplanted with donors yielding a low number of PIRCHE-II. To achieve this, PIRCHE-II should be directly implemented into the donor-selection criteria. However, implementing a prediction algorithm into the donor-selection criteria of organ exchange organizations is challenging, as implementation of an algorithm may have consequences on the well-balanced character of the currently used allocation systems. For example, changing the allocation algorithm may also impact additional factors such as time on waiting list, equality, and the kidney exchange rate between countries. Therefore, additional studies are required to investigate these aspects before implementing additional prediction algorithms into the donor-selection criteria. To study the potential impact of PIRCHE-II implementation into donor-selection criteria, we performed a simulation study of the Eurotransplant Kidney Allocation System (ETKAS). In this simulation study, ETKAS was simulated and HLA-related factors that are currently used in ETKAS were replaced by PIRCHE-II scores in different manners. Preliminary results show that the ETKAS can be simulated and that implementation of PIRCHE-II into ETKAS is feasible and does not affect additional factors such as waiting list time and the kidney exchange rate between countries (Niemann et al., manuscript in preparation).

**Fig. 1:**
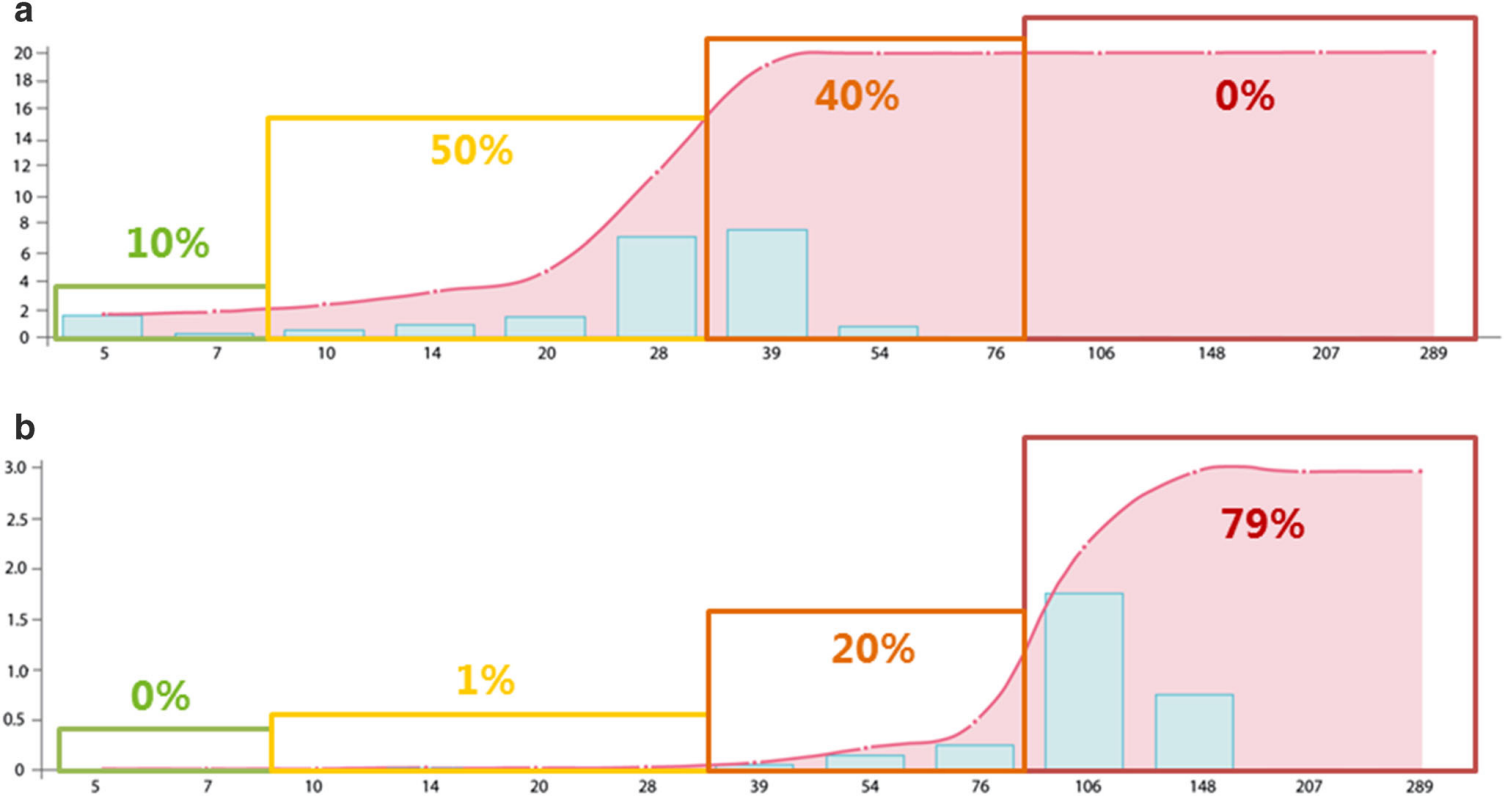
Estimating the probability of finding a donor with low PIRCHE-II via the SOT RAMP module. SOT RAMP histogram graphs are shown of two patients. The *x*-axes represent the number of PIRCHE-II, whereas the *y*-axes represent the corresponding approximation of getting a donor for a specific patient. The blue bars indicate approximation of getting a donor for a certain PIRCHE-II range. The accumulated approximation is indicated by a red line. The colored squares indicate low (green), low-intermediate (yellow), intermediate (orange), and high PIRCHE-II (red) and the percentages reflect the percentage of potential donors in the different PIRCHE-II groups. The histogram graph of patient A (**a**), shows that probability of finding a donor with low PIRCHE-II is high for this patient. In contrast, the histogram graph of patient B (**b**) shows that probability of finding a donor with low PIRCHE-II is very low for this patient

Since the number of PIRCHE-II not only is determined by the HLA of the donor, but also highly depends on the HLA background of the recipient, selecting a donor with low number of PIRCHE-II may not be feasible for every patient. To investigate this aspect for individual recipients, the Solid Organ Transplantation Risk and Acceptable Mismatch Profile (SOT RAMP) was developed (The SOT RAMP module is available via https://www.pirche.com). In the SOT RAMP module, individual recipients can be entered who are subsequently matched with a virtual deceased donor population (*n* = 10,000 virtual donors) using a preferred allocation search profile. The virtual deceased donor population has been generated based on HLA haplotype frequency tables from the National Marrow Donor Program. Four different ethnic virtual deceased donor populations can be used to match with an individual recipient. Moreover, the module also allows to upload custom populations, which can be used as a deceased donor population. After matching a recipient with a chosen allocation search profile, the module calculates the median PIRCHE-II that is achieved for that specific recipient in combination with the potential virtual donors. This PIRCHE-II median gives more insight into whether selecting a donor with a low number of PIRCHE-II is feasible or not. Also, histogram graphs are generated that display the approximation of getting a donor with a certain PIRCHE-II value. Two histogram graph examples are displayed in Fig. 1. For example, both patient A (Fig. 1a) and patient B (Fig. 1b) receive a kidney offer that yield a PIRCHE-II of 76. The histogram graph of patient A shows that the majority of the kidneys that theoretically can be offered to this patient has PIRCHE-II below 76. Thus, for this patient probability of finding a donor with low PIRCHE-II is high. In contrast, the histogram graph of patient B shows that the majority of the kidneys that theoretically can be offered to this patient have PIRCHE-II above 76. For this patient, the probability of finding a donor with low PIRCHE-II is very low. Thus, via these histogram graphs, clinicians can estimate whether a specific organ offer for a specific patients is an optimal organ offer in terms of PIRCHE-II. Additionally, the SOT RAMP module also indicates for each recipient which specific mismatches will lead to a high PIRCHE-II. By providing this mismatch information, the module also indicates for each recipient which HLA mismatches are preferably avoided based on the number of PIRCHE-II. Recently, the SOT RAMP module has been improved by adding allele frequency data to the acceptable mismatch profile and the possibility to add single antigen bead assay analyses of the recipient into the module. With these additions, clinicians cannot only see which mismatches will lead to a certain PIRCHE-II number, but also the frequency of that HLA in the general population and whether a recipient has HLA antibodies developed towards the HLA mismatches. Thus, this module allows clinicians to define the most optimal mismatch for their patients. Identification of HLA mismatches to which a recipient has [1] no HLA antibodies formed and [2] to which a recipient will not likely form HLA antibodies against after transplantation will allow the identification of acceptable mismatches at a next level.

## Concluding remarks

With the rise of next-generation sequencing, thousands of new HLA alleles were identified over the last few years (Abraham et al. 2018; Robinson et al. 2015; Robinson et al. 2016). Since the HLA system became extremely complex with regard to the ever-increasing number of identified HLA alleles, assessing the immunogenicity of individual HLA mismatches in vivo or in vitro became practically impossible. Algorithms appeared to be a practical solution to predict the immunogenic risk of individual HLA mismatches in solid organ transplantation. In this review, we focused on PIRCHE-II, a prediction algorithm for indirect T cell alloreactivity in solid organ transplantation settings. A higher number of PIRCHE-II was related to both de novo donor-specific HLA antibody formation and allograft rejection. These observations suggest that preferably one should transplant only with low numbers of PIRCHE-II. However, assessing the immunogenic potential of HLA mismatches by calculating the number of PIRCHE-II alone is not sufficient to predict whether the recipient will develop HLA antibodies and subsequent allograft rejection will occur or not. For example, transplanting a kidney with high numbers of PIRCHE-II does not guarantee that de novo donor-specific HLA antibodies will be formed after transplantation. PIRCHE-II should always be considered in the context of other factors. For example, recipient-related factors such as immunological status, immunosuppressive treatment and compliance to therapy, organ transplantation-related factors such as organ type and cold-ischemia time, and additional immunological factors such as the number of HLAMatchmaker eplets may also contribute to HLA antibody formation and subsequent allograft rejection. Furthermore, PIRCHE-II may theoretically also trigger regulatory T cell responses rather than alloreactive responses. Up to date, no studies have been performed to investigate the impact of PIRCHE-II on regulatory immune responses. Moreover, the PIRCHE-II algorithm itself requires further development to more precisely assess the immunogenic potential of HLA mismatches. Over the last few years, peptide-binding prediction algorithms have been improved (Andreatta et al. 2015; Karosiene et al. 2013), allowing a reasonably well prediction of peptide-binding. When these algorithms will be further improved, also the PIRCHE-II predictions will be further improved, allowing a better estimation of the immunological risk for individual solid organ transplantation recipients.
